# Postpartum hemorrhage care bundles to improve adherence to guidelines: A WHO technical consultation†

**DOI:** 10.1002/ijgo.13028

**Published:** 2019-12-23

**Authors:** Fernando Althabe, Michelle N.S. Therrien, Veronica Pingray, Jorge Hermida, Ahmet M. Gülmezoglu, Deborah Armbruster, Neelima Singh, Moytrayee Guha, Lorraine F. Garg, Joao P. Souza, Jeffrey M. Smith, Beverly Winikoff, Kusum Thapa, Emmanuelle Hébert, Jerker Liljestrand, Soo Downe, Ezequiel Garcia Elorrio, Sabaratnam Arulkumaran, Emmanuel K. Byaruhanga, David M. Lissauer, Monica Oguttu, Alexandre Dumont, Maria F. Escobar, Carlos Fuchtner, Pisake Lumbiganon, Thomas F. Burke, Suellen Miller

**Affiliations:** ^1^ Department of Mother and Child Health Institute for Clinical Effectiveness and Health Policy Buenos Aires Argentina; ^2^ Department of Reproductive Health and Research WHO Geneva Switzerland; ^3^ Safe Motherhood Program University of California San Francisco CA USA; ^4^ HERHealthEQ New York NY USA; ^5^ Quality Performance Institute University Research Co., LLC Chevy Chase MD USA; ^6^ Maternal and Newborn Division USAID Global Health Bureau Washington DC USA; ^7^ Department of Mother and Child Welfare Indian Institute of Health and Family Welfare Hyderabad India; ^8^ Division of Global Health and Human Rights Massachusetts General Hospital Boston MA USA; ^9^ Universidade de São Paulo São Paulo Brazil; ^10^ Jhpiego Baltimore MD USA; ^11^ Gynuity New York NY USA; ^12^ Maternal Child Survival Program and Maternal Health Jhpiego Washington DC USA; ^13^ International Confederation of Midwives Montréal QC Canada; ^14^ Bill and Melinda Gates Foundation Seattle WA USA; ^15^ Research in Childbirth and Health Group University of Central Lancashire Preston UK; ^16^ Department of Quality and Safety in Healthcare Institute for Clinical Effectiveness and Health Policy Buenos Aires Argentina; ^17^ Division of Obstetrics & Gynaecology St George's University of London London UK; ^18^ Department of Obstetrics & Gynecology Martyr's Hospital Ministry of Health Ibanda Uganda; ^19^ Institute of Metabolism and Systems Research University of Birmingham Birmingham UK; ^20^ Kisumu Medical and Education Trust Kisumu Kenya; ^21^ Research Institute for Sustainable Development CEPED Paris France; ^22^ Department of Gynecology and Obstetrics Fundacion Valle del Lili Cali Colombia; ^23^ FIGO Viador Pinto Bolivia; ^24^ Department of Obstetrics and Gynecology Faculty of Medicine Khon Kaen University Khon Kaen Thailand; ^25^ T.H. Chan School of Public Health, and HSPH Department of Global Health and Population Harvard Boston MA USA; ^26^ Department of Obstetrics, Gynecology & Reproductive Sciences Bixby Center for Global Reproductive Health and Policy University of California, San Francisco (UCSF) San Francisco CA USA

**Keywords:** Aortic compression, Bimanual compression, Intrauterine balloon tamponade, Non‐pneumatic antishock garment, Obstetric hemorrhage, Patient care bundles, Postpartum hemorrhage, Tranexamic acid, Uterotonics

## Abstract

**Objective:**

To systematically develop evidence‐based bundles for care of postpartum hemorrhage (PPH).

**Methods:**

An international technical consultation was conducted in 2017 to develop draft bundles of clinical interventions for PPH taken from the WHO's 2012 and 2017 PPH recommendations and based on the validated “GRADE Evidence‐to‐Decision” framework. Twenty‐three global maternal‐health experts participated in the development process, which was informed by a systematic literature search on bundle definitions, designs, and implementation experiences. Over a 6‐month period, the expert panel met online and via teleconferences, culminating in a 2‐day in‐person meeting.

**Results:**

The consultation led to the definition of two care bundles for facility implementation. The “first response to PPH bundle” comprises uterotonics, isotonic crystalloids, tranexamic acid, and uterine massage. The “response to refractory PPH bundle” comprises compressive measures (aortic or bimanual uterine compression), the non‐pneumatic antishock garment, and intrauterine balloon tamponade (IBT). Advocacy, training, teamwork, communication, and use of best clinical practices were defined as PPH bundle supporting elements.

**Conclusion:**

For the first response bundle, further research should assess its feasibility, acceptability, and effectiveness; and identify optimal implementation strategies. For the response to refractory bundle, further research should address pending controversies, including the operational definition of refractory PPH and effectiveness of IBT devices.

## Abstract 2

Care bundles were defined for first response and refractory PPH. Implementation strategies, refractory PPH definition, and effectiveness of intrauterine balloon tamponade require further consultation.

## 1 INTRODUCTION

Postpartum hemorrhage (PPH) occurs in approximately 5% of all live births and, despite concentrated efforts, remains a leading cause of maternal morbidity and mortality.^1^ Because most PPH‐related deaths are preventable through the implementation of effective interventions, the recent shift from home births to facility births across low‐ and middle‐income countries (LMIC) raises new opportunities for saving women's lives.^2^, ^3^ Unfortunately, inconsistent and/or delayed use of effective interventions for prevention and treatment of PPH, in addition to other systemic problems in health services (e.g., lack of blood banks, inadequate staffing), has led to continued unacceptable rates of hemorrhage‐related maternal deaths.^4^, ^5^, ^6^

Care bundles have been associated with improved patient outcomes when adherence is high.^7^, ^8^, ^9^ The concept of care bundles is similar to that of packages and checklists, which have been used by healthcare providers for decades with a similar goal of standardizing and expediting care (Supplementary Box S1). Care bundles may include behaviors, such as the widely used “ABCs” designed to help practitioners remember the sequence for resuscitation, or a number of interventions packaged together, such as the “Active Management of the Third Stage of Labor” (AMTSL) package used to prevent PPH.

In 2001, the Institute for Healthcare Improvement (IHI) developed a formal approach to bundling care to increase the quality and efficiency of care delivery.^10^ The IHI defined bundles as “small sets of evidence‐based interventions for a defined patient population and care setting that, when implemented together, result in significantly better outcomes than when implemented individually”.^10^ The “bundles” approach was designed to increase uptake and compliance to recommended interventions.^10^ Care bundles differ from other care packages in that compliance is achieved only when all the bundled interventions are completed and recorded. Thus, compliance for the bundle as a whole implies higher rates of compliance for its individual elements.^10^ Teamwork, communication, and cooperation are emphasized, because these health systems’ processes are required for quality and sustainability.^10^

In 2012, WHO published its “Recommendations for the Prevention and Treatment of Postpartum Haemorrhage” to provide evidence‐informed clinical care recommendations for hemorrhage due to uterine atony.^11^ However, adherence to these recommendations remains a challenge.^6^ The bundle approach has been proposed as a potential solution to suboptimal adherence to PPH guidelines.^4^ Healthcare bundles have been proposed for maternal conditions including placenta previa, elective induction, labor augmentation, vacuum delivery, maternal sepsis, and obstetric anal sphincter injury,^10^, ^12^, ^13^ but evidence of their success or failure is lacking. Although many current patient safety programs target PPH,^3^, ^5^, ^14^, ^15^, ^16^ there are no patient care bundles for PPH as defined by the IHI.

In early 2017, WHO decided to explore whether bundling current WHO‐recommended evidenced‐based interventions for PPH due to uterine atony might accelerate adoption and adherence to PPH guidelines. The aim of the present study was to describe the first steps toward that goal: the adoption of a bundle definition, the PPH intervention selection criteria, and the process for the development of two PPH care bundles.

## 2 MATERIALS AND METHODS

The consultation for the development of care bundles for PPH was carried out among international maternal health experts between October 2, 2017 and December 8, 2017. Completion of the online surveys and attendance at the in‐person meeting implied participant consent. The consultation did not require review by an institutional review board.

Postpartum hemorrhage was defined as bleeding that a skilled birth attendant (SBA) feels is excessive and worrisome for this exercise.^17^ In addition, in the absence of an accepted definition of refractory PPH, it was defined as bleeding that is unresponsive to initial treatment and that triggers an additional set of interventions.

**Figure 1:**
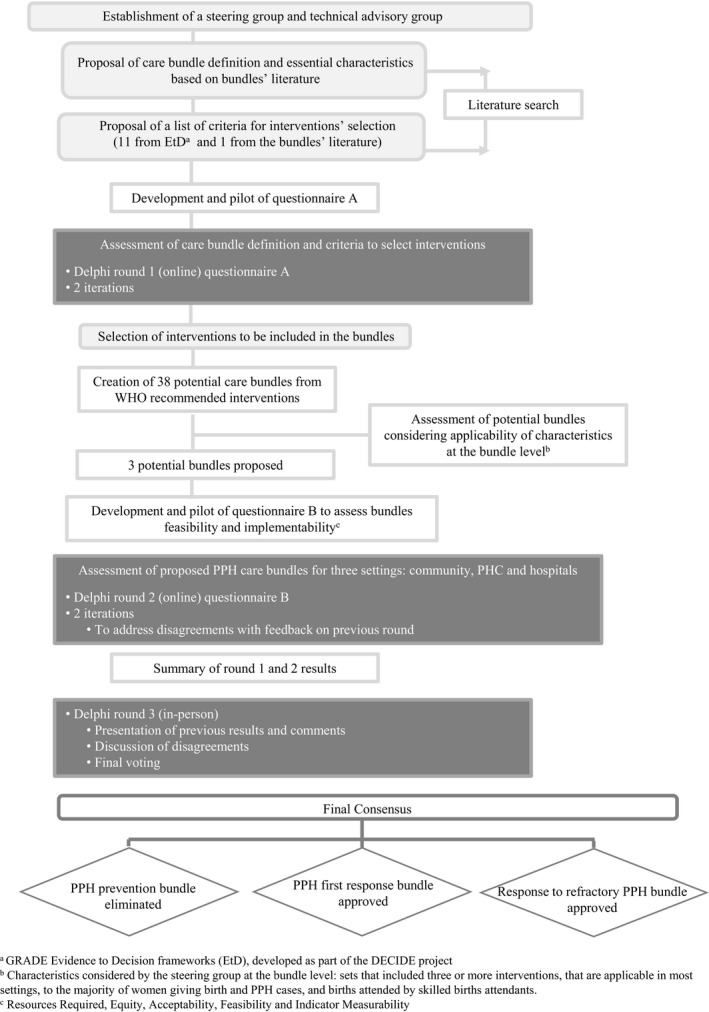
Flowchart of phases and procedures performed for the development of PPH care bundles during the technical consultation.

Development of the bundles was undertaken by a panel of experts with geographic and professional diversity (Supplementary File S1). The PPH bundles were developed first by conducting a systematic literature search to define care bundles and their essential characteristics in general; and then by identifying criteria to guide the selection of interventions for the PPH bundles. The selection of the interventions to be included in the bundles was made through technical consultations. Figure 1 outlines the process followed for bundle development.

The literature search was conducted by using PubMed, Medline, Cochrane Library, LILACS, WHO, PAHO, and Google to identify peer‐reviewed studies and grey literature (Supplementary File S2). Articles were included if they addressed the concept, development, and scientific evidence of patient care bundles in any field of medicine, with special attention to maternal healthcare and PPH bundles.

Regarding PPH bundles, the broad literature search initially looked at care bundles based on WHO recommendations and others. The interventions considered for inclusion in the PPH bundles were those specified in the 2012 WHO recommendations for hemorrhage due to uterine atony and the WHO 2017 update on tranexamic acid (TXA). To guide the selection of interventions, 11 criteria were selected from the validated and WHO‐adopted “GRADE Evidence to Decision” framework^18^ and one from the care bundle literature^10^, ^13^ (Table 1). For settings we considered community settings (i.e., home deliveries, health after delivery, and dispensary deliveries) assisted by SBAs, primary healthcare (PHC) centers, and hospitals. All WHO recommendations were assessed for appropriateness within each of these settings, resulting in 13 interventions eligible for inclusion (Table 2).

**Table 1:** Panel rating and agreement on the criteria used to assess the PPH care bundle

Order no.	Criterion	Description	Rating^a^	Agreement^b^
1	Desirable effects	How substantial are the desirable anticipated effects of the intervention? Judgments about how substantial the effects are should take into account the absolute magnitude of the effect (e.g., the proportion of individuals who would benefit) and the importance of the outcome (how much it is valued by the affected individuals)	8.5 (8–9)	Yes
2	Undesirable effects	How substantial are the undesirable anticipated effects of the intervention? Judgments about how substantial the undesirable effects are should take into account the absolute magnitude of the effect (e.g., the proportion of individuals who would benefit) and the importance of the outcome (how much it is valued by the affected individuals)	8 (7–8.5)	Yes
3	Certainty of the evidence on the effects	What is the overall certainty (also called quality) of the evidence of the intervention's effects? In the context of making decisions, the certainty rating reflects the extent of our confidence that the estimate of an effect (including test accuracy and associations) is adequate to support a particular selection	8.5 (7.5–9)	Yes
4	Values and preferences	Is there significant uncertainty about, or variability in, how much women value the outcomes associated with the intervention? Uncertainty about how much those affected (patients or their carers) value the outcomes of interest can be a reason for not selecting an intervention. Variability in how patients value the main outcomes (to the extent that individuals with different values would make different decisions) is another reason for not selecting an intervention	7 (4.5–7)	No
5	Balance of effects	Does the balance between desirable and undesirable effects favor the intervention? Judgments about the balance between the desirable and undesirable effects need to take into account the preceding four criteria: the magnitude of the desirable and undesirable effects, the certainty of the evidence supporting the anticipated effects, and how much those who are affected value the outcomes	8 (7–8.5)	Yes
6	Certainty of the evidence on resources required	What is the certainty of the evidence for the costs of the intervention? If resource use is considered critical for a recommendation, the less certain the evidence for resource requirements, the less likely it is that a panel should select or not the intervention	6 (4–7.5)	No
7	Cost‐effectiveness	Judgments about the cost effectiveness of an intervention need to take into account several criteria including the balance between the desirable and undesirable effects (the net benefit); the certainty of the evidence of effects and uncertainty about or variability in how much individuals value the main outcomes; and resource requirements (cost) and uncertainty about the costs	7.5 (7–8)	Yes
8	Resources required	How large are the resource requirements (costs in terms of both money and time) of the bundle? The greater the cost, the less likely it is that a bundle will be selected	8 (7.5–9)	Yes
9	Equity	What would be the impact of the bundle on health equity? This criterion evaluates if a bundle is expected to reduce health inequities. It considers whether a bundle will reduce differences in the effectiveness for disadvantaged populations within countries, such as low‐income groups, less educated individuals, and/or rural populations	8 (6.5–9)	Yes
10	Acceptability	Is the bundle acceptable to key stakeholders (women and providers)? A bundle might vary on its acceptability level due to ethical principles (e.g., autonomy, beneficence or justice), as well as the distribution of the desirable and undesirable effects and costs (who benefits or is harmed, and who pays or saves)	8 (6.5–9)	Yes
11	Feasibility	Is the bundle feasible to implement? Feasibility is influenced by factors such as the resources available, infrastructure, and training. If the bundle elements are not already in use, this criterion evaluates if the bundle can be introduced with a reasonable investment of cost, time, and training. Clinicians might find a care bundle unhelpful if the included interventions are not implementable in their settings	8 (7–8)	Yes
12	Indicator measurability	This criterion evaluates whether an indicator for the intervention's use is available and can be simply and reliably measured during routine clinical practice, without or with a minimum of extra resources. Indicators are quantitative or qualitative factors or variables that provide a simple and reliable means to measure achievement	5 (5–7)	No

a Values are given as median (interquartile range).

b Agreement was defined with as a disagreement index of <1.

**Table 2:** Description of WHO‐recommended clinical interventions for PPH, 2012–2017

Intervention	Description
Uterotonics	Administration of oxytocin (IV/IM); ergometrine/methylergometrine or other fixed drug combination of oxytocin and ergometrine (IM); misoprostol (oral).The preferred drug for prevention of PPH is oxytocin (10 IU, IV/IM). If unavailable, give IM ergometrine/methylergometrine or the fixed drug combination of oxytocin and ergometrine, if not contraindicated. If IM or IV uterotonics are unavailable, give oral misoprostol (600 μg)
Controlled cord traction	After delivery of the newborn and it is assessed that there are no other fetuses in utero, gentle traction is applied to the umbilical cord with one hand, while the other hand applies abdominal counter‐pressure on the uterus
Postpartum abdominal uterine tonus assessment	Palpate the uterus to assess uterine firmness/tone; if the uterus is soft or flabby this may indicate uterine atony
Isotonic crystalloids	Administration of a starting dose: 500 mL of isotonic crystalloids IV, in 30 min; and continuing doses of 500 mL of isotonic crystalloids IV, in 60 min
TXA	A fixed dose of 1 g of TXA (100 mg/mL IV at 1 mL per min), within 3 h of the time of diagnosis (if unknown, time of delivery); a second dose of 1 g can be given if needed 30 min after the first dose
Uterine massage	Circular rubbing of the uterus achieved via manual massaging of the abdomen. This is typically sustained until the bleeding stops or the uterus contracts
Intrauterine balloon tamponade	The procedure entails insertion of a deflated/uninflated balloon into the uterine cavity and then inflating it to achieve a tamponade effect
Bimanual uterine compression	Two handed, one in the anterior vaginal fornix and one behind the uterine fundus, squeezing the uterus between the hands
External aortic compression	External compression applied with a closed fist at the level of the umbilicus and slightly to the woman's left
NASG	Used as a temporizing measure until source of bleeding found and treated. NASG is a lower body compression device made of stretch neoprene which closes tightly with Velcro in segments for the ankles, calves, thighs, pelvis, and abdomen and is applied rapidly starting at the ankles
A single dose of antibiotics	In the context of placental retention, the placenta should be extracted, and a single dose of antibiotics administered
Uterine artery embolization	If other measures have failed and if the necessary resources are available, the use of uterine artery embolization is recommended as a treatment for PPH due to uterine atony
Surgical intervention	If bleeding persists despite treatment with uterotonic drugs and other conservative interventions, surgical intervention should be used without further delay

Abbreviations: IM, intramuscular; IV, intravenous; NASG, non‐pneumatic antishock garment; PPH, postpartum hemorrhage; TXA, tranexamic acid.

The 13 interventions were then classified according to purpose (prevention, first response, and response to refractory PPH); setting (as above); application to vaginal delivery, cesarean delivery, or any type of delivery; and application during the third stage of labor or the first 24 hours postpartum. From a total of 38 possible combinations of the 13 interventions that emerged from the above classification, those that included three or more interventions, were judged to be applicable in most settings, were intended for use by skilled birth attendants, and would be applicable to most women with PPH due to uterine atony were selected. Two recommended interventions, hemostatic surgery and arterial embolization, were excluded from the bundles because neither is feasible in most settings nor applicable to most women with PPH due to uterine atony.

The definition of care bundles, the criteria for selecting interventions, and the potential PPH care bundles were agreed upon by the experts through iterative consensus exercises. The process used a three‐stage modified Delphi method,^19^ starting with two rounds of individual and anonymous online questionnaires with closed‐ended and open‐ended questions, followed by a third round which was an in‐person technical consultation. The first round began with questionnaire A (Supplementary File S3), which focused on the definition of patient care bundles and criteria to guide design of the bundles, followed by questionnaire B (Supplementary File S4), which asked the expert panel to provide relevance ratings (on a 1–9 Likert scale, where 7–9 was considered a “high median relevance rating”) of the individual bundles in relation to feasibility and implementability for three different settings. (Supplementary File S3). Each questionnaire underwent two rounds, the results of which provided inputs (median relevance rates and comments) toward consensus. Consensus was based on the ratings distribution in accordance with the RAND/UCLA criteria.^20^

The experts met for an in‐person consultation December 7–8, 2017, to consolidate agreements and to address disagreements. Presentations and discussions were held in plenary sessions, where the “poll everywhere” audience response system and paper ballots were used to record individual decisions anonymously. See Supplementary File S5 for details.

## 3 RESULTS

In the literature search, 730 articles met the initial criteria, of which 415 were excluded after reviews of the abstract and full text (Supplementary Fig. S1). Informed by the literature review, the experts developed the following definition of patient care bundles, adapted from the IHI definition^10^ with input from Lagan: “a patient care bundle is a limited set of evidence‐based interventions for a defined patient population and care setting, procedure, or treatment” (from personal communication with Sally Lagan, National Special Projects Manager in 2003). Care bundles are meant to organize and simplify patient care, reinforce team performance, increase adherence to recommendations, and reduce variability. Some characteristics that make bundles unique include their limited number of interventions (3–5 elements), the fact that the bundle is not a decision‐making algorithm or checklist, and the fact that measurement of compliance during implementation is based on the use of all interventions.^10^ [Correction added on 21 January 2020, after first online publication: (3‐5) was removed from superscript.]. The definition and characteristics of care bundles were approved by the experts in the first online consultation. The systematic literature search also helped to describe different types of bundles and the interventions that are included in bundles, as well as to identify studies that describe PPH care bundles (Supplementary [Link], [Link]).

Table 1 summarizes the 12 criteria agreed upon to assess PPH care bundles, their definition, median relevance rates, and the level of agreement in accordance with RAND relevance ratings (Supplementary File S3). The experts did not suggest additional criteria. There was no agreement on the relevance ratings of the following criteria: values and preferences, certainty of the evidence of resources, and indicator measurability due to divergent opinions. The other criteria received high relevance ratings (median rating 7–9).

Three PPH care bundles that met the agreed criteria were initially identified: (1) prevention and recognition of PPH; (2) first response to PPH; and (3) response to refractory PPH. Among these three bundles, one was rejected and two were accepted.

### 3.1 Prevention and recognition of PPH bundle

The bundle of interventions proposed for PPH prevention included uterotonics, controlled cord traction (CCT), and uterine tone assessment. In the online rounds, this bundle received high relevance rates and strong agreement overall (Supplementary Table S3). However, several issues emerged during the online rounds and were discussed at the in‐person meeting.

The experts agreed that the proposed bundle of interventions was very similar to the Active Management of the Third Stage of Labor package, which in recent years has been de‐emphasized as a care package by the WHO. One of the elements, CCT, was recently demonstrated to have little effect on PPH,^21^ and was only recommended conditionally in the 2012 recommendations. The expert panel agreed that bundle compliance and compliance measurement would be affected by conditional application of CCT; therefore, a bundle should not be developed for prevention of PPH, and care should continue as recommended independent interventions.

### 3.2 First response to PPH bundle

The set of interventions proposed for the first response to PPH care bundle included uterotonics, intravenous (IV) isotonic crystalloids, TXA, and uterine massage. During the online rounds of consultation, this bundle received high relevance rates and agreement from the experts for implementation at the PHC and hospital levels. However, the group expressed concerns that there might be barriers to implementation in many community settings, and the bundle might require a substantial amount of resources, such as equipment, supplies, training, health policies, and regulations.

The group approved the bundle for the treatment of PPH due to uterine atony in hospitals and PHCs, and in the community if implemented by an SBA who was appropriately equipped and trained. The expert panel suggested acronyms that might be used for this bundle such as “MOTIVate” or “MOTIV8,” meaning massage, oxytocics, TXA, and IV fluids.

### 3.3 Response to refractory PPH bundle

The following set of interventions was proposed for the response to refractory PPH care bundle: continue administration of uterotonics and isotonic crystalloids, second dose of TXA, IBT, and non‐pneumatic antishock garment (NASG). It was acknowledged that IBT or NASG may not be available in some settings.

During the online consultation, this bundle, intended for women who continue to bleed despite implementation of the first response bundle and whose condition worsens or deteriorates, received high RAND relevance scores, and had the agreement of the panel for the PHC and hospital levels. For the community level, however, the bundle received low RAND relevance scores for four criteria (acceptability, feasibility, indicator measurability, and no or minimal resources required), and there was no consensus for the equity criteria (Supplementary Table S3).

During the discussions at the in‐person meeting, the following issues were discussed for the refractory bleeding bundle. First, uterotonics, crystalloids, and TXA were already included in the first response bundle, and therefore did not need to be listed as bundle components. Second, IBT is currently recommended by the WHO, but is considered controversial by some members owing to recently published evidence.^22^, ^23^, ^24^, ^25^ Third, in cases where IBT or NASG is not available, or for use during the period before IBT and NASG are applied, bimanual uterine compression and external aortic compression were suggested for bundle inclusion by some experts. Fourth, concerns were raised that implementing all elements of the bundle might result in the overtreatment of women with refractory hemorrhage whose condition was not worsening. Last, an area of contention was whether or not the “response to refractory PPH bundle” should be a bundle. Some members mentioned that the conditional, variable, and progressive changes of refractory hemorrhage may make this condition less appropriate for the bundle approach.

In response to these concerns, the panel considered the following points: (1) that the interventions from the first response bundle should be removed from the refractory bundle (uterotonics, crystalloids, and TXA); (2) that new evidence would continue to arise about all interventions in the bundles, and thus all interventions would be reconsidered by the WHO for inclusion in their future recommendations^26^; and (3) that the initial, agreed‐upon assumption had been to define refractory hemorrhage as bleeding that is resistant to first response measures and is accompanied by worsening maternal condition. Some experts proposed creating a refractory PPH care package with all recommended interventions, but allowing for adaptation dependent on local conditions, as an alternative to the response to refractory PPH care bundle; however, this idea was not accepted by most experts.

The panel's final decision was to support the refractory bundle summarized in Box 1, comprising two manual compressive measures (aortic or bimanual uterine compression) and two devices, IBT and NASG, acknowledging that care providers may not implement the full bundle if the hemorrhage stops after one or some of the interventions. The primary rationale for keeping these interventions in a bundle was, first, that the “care package” approach has been recommended by WHO since 2012; and second, the rationale for proposing a bundle approach was to improve strategies for compliance with best practices.

Box 1 **Final care bundles for postpartum hemorrhage.**

**First response PPH bundle**

Uterotonic drugs

Isotonic crystalloids

Tranexamic acid

Uterine massage

Notes: Initial fluid resuscitation is performed together with intravenous (IV) administration of uterotonics. If IV uterotonics are not available, fluid resuscitation should be started in parallel with sublingual misoprostol or other parenteral uterotonics. If postpartum hemorrhage (PPH) is in the context of placental retention, the placenta should be extracted and a single dose of antibiotics should be administered.

**Response to refractory PPH bundle**

Compressive measure (aortic compression or bimanual uterine compression)

Intrauterine balloon tamponade

Non‐pneumatic anti shock garment

Notes: A continuing dose of uterotonics (e.g., oxytocin diluted in isotonic crystalloids) and a second dose of tranexamic acid should be administered during the application of this bundle.

The original aim was that the PPH bundles would apply to both vaginal and cesarean delivery; however, additional discussions made it clear that post‐cesarean bleeding might require a modified approach for the following reasons: uterine massage may not be effective for these women; uterotonics and IV fluids are likely to be already in place, making these two components of the first response bundle redundant for most patients; and the early detection of PPH is likely to use different strategies as compared with vaginal delivery. Therefore, the opinion of the group was that a modified bundle that addresses the unique circumstances and needs of post‐cesarean bleeding should be developed and evaluated.

The panel additionally advised that the two bundles are not meant to reflect comprehensive clinical care and that best clinical practices must be observed (Supplementary File S5). Lastly, the expert panel agreed that the bundle development process had focused on current WHO recommended interventions. These PPH bundles are “living bundles” and will be re‐examined as new evidence emerges during the process of updating WHO recommendations and guidelines.^26^

## 4 DISCUSSION

In the present consultation, a systematic approach was used to review the care bundle literature to develop care bundles for atonic PPH after vaginal delivery, the elements of which were based on WHO‐recommended PPH interventions.^1^, ^11^ The definition of a patient care bundle was adapted from the IHI bundle definition as “a limited set of evidence‐based interventions for a defined patient population and care setting, procedure, or treatment.” Through online and face‐to‐face consultations, a group of PPH experts came to consensus on a PPH first response bundle, consisting of uterotonics, isotonic crystalloid IV fluids, uterine massage, and TXA, for implementation at both the PHC and hospital levels. The discussion around the response to refractory PPH bundle, which included bimanual uterine compression, aortic compression, IBT, and NASG, in addition to continuing with IV fluids, uterotonics, and TXA raised some controversy, although the majority of the group was in agreement about adopting it as a bundle.

The consultation process has several strengths. In the absence of a validated method for bundle development, a methodologically rigorous, transparent, and reproducible process was developed for the design of the care bundles. This process included a comprehensive literature review, a well‐accepted and recommended list of evidence‐based interventions, a previously validated framework of criteria to guide the selection of WHO‐recommended interventions for atonic PPH for the bundles, and a consensus development process among experts using the accepted modified Delphi technique.

However, there were limitations to the process. First, inherent to any consensus process is bias due to the influence of interpersonal dynamics. We tried to ameliorate this by having a diverse panel of clinical and academic experts balanced by gender, region, and profession, and by the anonymity of the online consultations; in addition, all members completed the disclosure of interest form required by WHO. Second, the process to modify and accept the response to refractory PPH bundle at the in‐person meeting was different from the consensus protocol used for the online consultations. Last, since the publication of the 2012 WHO PPH recommendations, only one intervention has been updated (TXA in 2017). It is possible that new evidence may result in changes to the recommendations.

The two proposed PPH bundles may warrant different approaches in the next stages of development. The first response PPH bundle fulfills the characteristics and criteria of a care bundle, as articulated by the IHI. It includes four recommended interventions, agreed upon without exception, which should all be administered to all women with PPH due to uterine atony.

By contrast, the issues raised about the response to refractory PPH may merit further analyses and discussions. Although several publications have reported positive outcomes with IBTs,^23^, ^24^, ^25^ a randomized controlled trial reported safety concerns associated with implementation of a condom catheter IBT.^22^ Similarly, preliminary results of a stepped‐wedge cluster randomized trial in Egypt, Senegal, and Uganda raised safety concerns associated with the implementation of an improvised condom catheter IBT for treatment for unresponsive PPH (based on communication from the Gynuity Health Projects research team received on 2/8/2018). To our knowledge, these studies are the only randomized controlled trials of improvised condom catheter IBTs versus no IBT. Furthermore, WHO updates on IBT recommendations will be released in 2019.

The panel agreed that the response to refractory PPH bundle was intended to treat critically ill women who continued to bleed despite first response measures and whose condition was worsening or deteriorating. However, this restricted definition may generate uncertainties for clinicians about how to treat women with refractory PPH whose condition remains initially stable. On the one hand, the bundle approach might be clinically less useful if a large proportion of women with refractory PPH are ineligible for bundle application. On the other hand, if all bundled interventions are given to all women with refractory PPH (as the bundle literature demands), there might be the potential to “overtreat” some women. It is acknowledged that many care providers will stop implementing other bundle components if the initial intervention works; however, that approach, even if clinically logical, would contradict the accepted definition of a “care bundle,” in which all interventions should be administered. If not all interventions are administered, the response to refractory PPH “bundle” would be more similar to a care package, where a clinical algorithm is used to define which interventions to apply and when to stop.^28^ Many of the experts were more concerned about undertreatment and delayed recognition of PPH than about the risk of overtreatment of women with severe refractory PPH. Experts raised the issue of the impossibility of a single front‐line worker being able to perform all of the bundle interventions if they were applying either of the manual compression measures. In addition, some experts stated that it was possible that the clinical conditions of women experiencing refractory PPH might be too variable, progressive, and conditional, thereby requiring an incremental, more tailored, individualized approach rather than a care bundle approach.

The development and implementation of the bundles should not prevent care providers from making a thorough assessment of the etiology of PPH before intervening. We note that, although both TXA and the NASG can be effective for non‐atonic obstetric hemorrhage etiologies, these bundles are recommended for uterine atony. Although the proposed care bundles are based on rigorously developed evidence‐based recommendations, they have yet to be tested and evaluated as a strategy to improve clinical care for PPH.

For the first response PPH bundle, the next phase is the development of an implementation strategy, culminating in a model for use at the facility level in LMICs. This strategy must include training on use of the bundles; support for health systems’ processes of communication, teamwork, and cooperation; packaging bundles with non‐commodity components; and supportive supervision, monitoring, and evaluation.

For the response to refractory PPH bundle, it is a priority to solve pending controversies including the operational definition of refractory PPH, and to better understand the effectiveness of various IBT devices. For any PPH bundle, strengthening commodity supply chains and encouraging behavior change are critical to implementation. Assessment of facilitators and barriers should guide the development of the strategy. The approach will need to be tailored to local contexts to ensure sustainability. Similarly, leadership from ministries and key stakeholders will be critical for successful bundle implementation. We expect that the PPH bundles will reduce rates of severe PPH, morbidity, and mortality, through improved quality of care and adherence to global, high‐quality guidelines; however, this has not yet been demonstrated. Future research must rigorously assess how these bundles are implemented in practice, including the mechanisms of impact and how these are influenced by the context.^28^ [Correction added on 21 January 2020, after first online publication: the reference citation 29 was changed to 28.] Factors to be evaluated include bundle feasibility, acceptability, safety, adverse consequences, and effectiveness relative to individual interventions. The opinions of healthcare planners, practitioners, and users will be important to consider. Cost‐effectiveness and impact should be studied at both the hospital and PHC levels to evaluate the value of the bundles in different settings and relative to other strategies, which might better improve use of recommended individual interventions. Although both bundles are suitable for use in PHCs, early adoption and ownership at the referral hospitals in their catchment area will build support for introduction into PHCs; therefore, an incremental introduction may be necessary. Because the expert panel developed PPH bundles for facility‐level implementation, other strategies may need to be developed for deliveries taking place at the community level. There also may need to be consideration of what bundle elements may be implemented if there is only one provider (with one pair of hands).

Given these considerations, there will be a need for implementation research to determine if the bundling approach will ultimately make a difference in saving women's lives from PPH.

## AUTHOR CONTRIBUTIONS

Study conceptualization: TFB, JL, SM, and JPS. Systematic search and data analysis: FA, EGE, VP, and MST. Survey development: FA, EGE, SM, VP, JPS, and MST. Manuscript writing: FA, TFB, EGE, LFG, MG, AMG, JL, SM, VP, JPS, and MST. Participation in technical consultations: DA, FA, EB, TFB, AD, SD, EGE, MFE, CF, LFG, MG, EH, JH, DL, JL, PL, MO, SM, VP, NS, JMS, JPS, MST, KT, and BW. Review and approval of manuscript: DA, FA, SA, EKB, TFB, AD, SD, EGE, MFE, CF, LFG, AMG, MG, EH, JH, DL, JL, PL, MO, SM, VP, NS, JMS, JPS, MST, KT, and BW.

## CONFLICTS OF INTEREST

The authors have no conflicts of interest. SM received funds from Blue Fuzion. JMS received grants from USAID during the study. The views expressed in the study do not necessarily reflect the views of the WHO, USAID, or the US Government.

## Supporting information


**Figure S1.** Flowchart of the systematic search of the literature.

**Table S1.** Type of bundles and interventions based on the systematic search of the literature.

**Table S2.** Main characteristics of the maternal and PPH studies reviewed.

**Table S3.** Performance of each bundle for the five feasibility criteria assessed at the three individual settings.

**File S1.** Technical consultation participants.

**File S2.** Methodologic details.

**File S3.** Survey 1.

**File S4.** Survey 2.

**File S5.** Technical consultation agenda.

**Box S1.** Examples of best clinical practices**.**
