# Supplementary material for: Postpartum hemorrhage care bundles to improve adherence to guidelines: A WHO technical consultation
Source: Int J Gynaecol Obstet. 2019 Dec 23;148(3):290–9. doi: 10.1002/ijgo.13028 (PMC7064978; doi:10.1002/ijgo.13028)
Supplement: Supplementary file 1 — Figure S1. Flowchart of the systematic search of the literature. [file IJGO-148-290-s001.docx]

##

## **Supplementary Figure S1** Flowchart of the systematic search of the literature

Articles removed:

- Payment bundles: (n=71)

-Non English or Spanish articles: (n=11)

-Duplicate (n=15)

-Other topic (n=11)

- No full text (n=28)

Articles screened by full text
(n= 594)

Full Text Articles excluded **(n=34)** due to:

-editorial, case study, protocol, comment or letter

Total articles identified

PubMed

**(n= 730)**

Articles assessed by eligibility

(n =560)

Full text articles excluded (n=245) due to:

- Bundle development not reported (n=45)

- Bundle definition not reported (n=30)

-No care bundle (n=170)

Total number of articles included in the review

(n =315)
