# Supplementary material for: Postpartum hemorrhage care bundles to improve adherence to guidelines: A WHO technical consultation
Source: Int J Gynaecol Obstet. 2019 Dec 23;148(3):290–9. doi: 10.1002/ijgo.13028 (PMC7064978; doi:10.1002/ijgo.13028)
Supplement: Supplementary file 2 — Table S1. Type of bundles and interventions based on the systematic search of the literature. [file IJGO-148-290-s002.docx]

## **Supplementary Table S1** Type of bundles and interventions based on the systematic search of the literature

| Type of bundle | Type of interventions | | | Total  n (%) |
| --- | --- | --- | --- | --- |
|  | Clinical  n (%) | Clinical & Organizational  n (%) | Organizational  n (%) |  |
| Maternal and Perinatal Bundles |  |  |  |  |
| Condition oriented | 4 (13) | 9 (29) | 1 (3) | 14 (45) |
| Procedure oriented | 9 (29) | 6 (19) | 2 (6) | 17 (55) |
| Total | 13 (42) | 15 (48) | 3 (10) | 31 (100) |
| Bundles in Other Areas of Care |  |  |  |  |
| Condition oriented | 69 (46) | 26 (17) | 4 (2) | 99 (66) |
| Procedure oriented | 25 (16) | 9 (6) | 18 (12) | 52 (34) |
| Total | 94 (62) | 35 (23) | 22 (14) | 151 (100) |
