# Supplementary material for: Postpartum hemorrhage care bundles to improve adherence to guidelines: A WHO technical consultation
Source: Int J Gynaecol Obstet. 2019 Dec 23;148(3):290–9. doi: 10.1002/ijgo.13028 (PMC7064978; doi:10.1002/ijgo.13028)
Supplement: Supplementary file 3 — Table S2. Main characteristics of the maternal and PPH studies reviewed. [file IJGO-148-290-s003.docx]

| **Supplementary Table S2** Main characteristics of the maternal and PPH studies reviewed. | | | |  |  |  |
| --- | --- | --- | --- | --- | --- | --- |
| Study | Country | Topic | Definition of Bundle | Orientation | Type of bundles | Proposed Approach |
| ***Maternal Care Bundles*** | | | | | | |
| IHI  2010 | US | Vacuum Assisted Delivery | IHI | Procedure | Clinical | Bundle |
| IHI  2011 | US | Elective Induction | IHI | Procedure | Clinical | Bundle |
| IHI  2012 | US | Augmentation Bundle | IHI | Procedure | Clinical | Bundle |
| Kendig  2017 | US | Perinatal Depression and Anxiety | Council on Patient Safety in Women's Health Care | Condition | Clinical and organizational | Bundle |
| Bernstein 2017 | US | Hypertension | Council on Patient Safety in Women's Health Care | Condition | Clinical and organizational | Bundle |
| Main  2015 | US | Obstetric Haemorrhage | Council on Patient Safety in Women's Health Care | Condition | Clinical and organizational | Bundle |
| D’Alton 2016 | US | Venous Thromboembolism | Council on Patient Safety in Women's Health Care | Condition | Clinical and organizational | Bundle |
| Bernstein 2017 | US | Reduction of Peripartum Racial/Ethnic Disparities | Council on Patient Safety in Women's Health Care | Social Determinants | Organizational | Bundle |
| ACOG  2014 | US | Safe Reduction of Primary Cesarean Birth | Council on Patient Safety in Women's Health Care | Procedure | Clinical and organizational | Bundle |
| Kawakita 2017 | US | Surgical Site Infection od Cesarean Delivery | IHI | Condition | Clinical | Bundle |
| Moroz  2016 | US | Severe Hypertension in Pregnancy | Does not apply | Condition | Clinical and organizational | Protocol, package and/or safety program |
| ACOG | US | Support After Severe Maternal Event | Council on Patient Safety in Women's Health Care | Procedure | Clinical and organizational | Bundle |
| ACOG | US | Severe maternal morbidity review | Council on Patient Safety in Women's Health Care | Procedure | Organizational | Bundle |
| ACOG | US | Postpartum care | Patient safety | Procedure | Clinical and organizational | Bundle |
| Rasmussen 2016 | Denmark | Obstetric Sphincter Injuries | Not defined | Condition | Clinical and organizational | Bundle |
| Tolcher 2016 | US | Labor and delivery care | Not defined | Procedure | Clinical and organizational | Bundle |
| Minkoff 2009 | US | Fetal monitoring bundle | IHI | Procedure | Organizational | Bundle |
| RCOG  2010 | UK | Electronic fetal monitoring | IHI | Procedure | Clinical | Bundle |
|  |  |  |  |  |  |  |
| ***Obstetrical/Postpartum Hemorrhage*** | | | | | | |
| Main  2015 | US | Obstetric Hemorrhage | Council on Patient Safety in Women's Health Care | Condition | Clinical and organizational | Bundle |
| RCOG  2010 | UK | Placenta praevia after cesarean section | IHI | Condition | Clinical and organizational | Bundle |
| Skupski 2006 | US | Obstetric hemorrhage | Does not apply | Condition | Organizational | Protocol, package and/or safety program |
| Shields  2011 | US | Maternal Hemorrhage | Does not apply | Condition | Clinical and organizational | Protocol, package and/or safety program |
| Shields  2015 | US | Obstetric Hemorrhage | Does not apply | Condition | Clinical and organizational | Protocol, package and/or safety program |
| Einerson 2014 | US | Postpartum Hemorrhage | Does not apply | Condition | Clinical and organizational | Protocol, package and/or safety program |
| Deneux-Tharaux 2011 | France | Severe Postpartum Hemorrhage | Does not apply | Condition | Clinical and organizational | Protocol, package and/or safety program |
| Rizvi  2004 | Ireland | Massive Postpartum Hemorrhage | Does not apply | Condition | Clinical | Protocol, package and/or safety program |
| Bajaj  2016 | US | Eclampsia and Postpartum Hemarrhage | Does not apply | Condition | Clinical and organizational | Protocol, package and/or safety program |
| Bingham 2011 | US | Obstetric Hemorrhage | Does not apply | Condition | Organizational | Protocol, package and/or safety program |
| Main  2017 | US | Obstetric hemorrhage | Council on Patient Safety in Women's Health Care | Condition | Organizational | Protocol, package and/or safety program |
| Lappen  2013 | US | Postpartum Hemorrhage | Does not apply | Condition | Clinical and organizational | Protocol, package and/or safety program |
| Lyndon 2015 | US | Obstetric hemorrhage | Council on Patient Safety in Women's Health Care | Condition | Clinical and organizational | Bundle and Toolkit |
