# Supplementary material for: Postpartum hemorrhage care bundles to improve adherence to guidelines: A WHO technical consultation
Source: Int J Gynaecol Obstet. 2019 Dec 23;148(3):290–9. doi: 10.1002/ijgo.13028 (PMC7064978; doi:10.1002/ijgo.13028)
Supplement: Supplementary file 4 — Table S3. Performance of each bundle for the five feasibility criteria assessed at the three individual settings. [file IJGO-148-290-s004.docx]

## **Supplementary Table S3** Performance of each bundle for the five feasibility criteria assessed at the three individual settings.

| Bundle | | Criteria | | | | | | Overall beneficial rating* |
| --- | --- | --- | --- | --- | --- | --- | --- | --- |
|  |  | Equity (ω_e_=8.79) | | Acceptability (ω_a_=8.79) | Feasibility (ω_f_=9.34) | Indicator Measurability (ω_i_=5.49) | Resources required (ω_r_=8.79) |  |
|  |  | Rating (partial weighted rating) | | | | | |  |
| **PPH Prevention and recognition** | |  |  | | | |  |  |
|  | For Community | 7 (62) | | 8 (70) | 7 (65) | 7 (38) | 7 (61) | 297 |
|  | For PHC | 8 (70) | | 8 (70) | 8 (75) | 8 (44) | 8 (70) | 330 |
|  | For Hospitals | 9 (79) | | 9 (79) | 8 (75) | 9 (49) | 8 (70) | 353 |
| **First Response** | |  | |  |  |  |  |  |
|  | For Community | 7 (62) | | 6.5 (57) | 3(28) | 7 (38) | 2 (18) | 203 |
|  | For PHC | 8 (70) | | 8 (70) | 7 (65) | 7 (38) | 6 (53) | 297 |
|  | For Hospitals | 7.5 (66) | | 9 (79) | 9 (84) | 8 (44) | 8 (70) | 343 |
| **Continuing Bleeding** | |  | |  |  |  |  |  |
|  | For Community | 6.5 (57) | | 3 (26) | 3 (28) | 4 (22) | 2 (18) | 151 |
|  | For PHC | 8 (70) | | 7 (62) | 7 (65) | 7 (38) | 5 (44) | 280 |
| For Hospitals | | 7 (62) | | 9 (79) | 9 (84) | 8 (44) | 7 (62) | 330 |

Note: ω represents each criteria individual weight

*Overall beneficial rating (weighted sum) = (rating_e_* ω_e_) _+_ (rating_a_* ω_a_) + rating* ω_f_) + (rating_i_* ω_i_) + (rating_r_* ω_r_)
