# Supplementary material for: Postpartum hemorrhage care bundles to improve adherence to guidelines: A WHO technical consultation
Source: Int J Gynaecol Obstet. 2019 Dec 23;148(3):290–9. doi: 10.1002/ijgo.13028 (PMC7064978; doi:10.1002/ijgo.13028)
Supplement: Supplementary file 5 — File S1. Technical consultation participants. [file IJGO-148-290-s005.docx]

# **Supplementary File S1** Technical Consultation Participants

## Lists of participants

| **Technical Advisory Group** |  |  |
| --- | --- | --- |
| Pisake Lumbiganon, Chair | |  |
| Alexandre Dumont |  |  |
| Beverly Winikoff |  |  |
| Carlos Fuchtner |  |  |
| David Lissauer |  |  |
| Deborah Armbruster |  |  |
| Emmanuel Byaruhanga |  |  |
| Emmanuelle Hébert |  |  |
| Jeffrey Smith |  |  |
| Jorge Hermida |  |  |
| Justus Hofmeyr |  |  |
| Kusum Thapa |  |  |
| Maria Escober |  |  |
| Monica Oguttu |  |  |
| Neelima Singh |  |  |
| Susan Downe |  |  |
| **Facilitator** |  |  |
| Jamie Higgins (non-voting) | |  |
| **Host** | |  |
| Wafaie Fawzi (non-voting) | |  |
| **Steering Group** | |  |
| WHO | |  |
| Joao Paulo Dias de Souza (non-voting) | |  |
| Bill and Melinda Gates Foundation |  | |
| Jerker Liljestrand (non-voting) | |  |
| Massachusetts General Hospital |  | |
| Thomas Burke | |  |
| UCSF |  | |
| Suellen Miller | |  |
| Michelle Skaer Therrien (non-voting) |  | |
| IECS |  | |
| Fernando Althabe | |  |
| Veronica Pingray (non-voting) |  | |
| Ezequiel García Elorrio (non-voting) |  | |
| **Observers (non-voting)** |  | |
| Alicia Lightbourne |  | |
| Ana Langer |  | |
| Daniela Suarez-Rebling |  | |
| Lori Garg |  | |
| Moytrayee Guha |  | |
| Mrunal Shetye |  | |
|  |  | |
