# Supplementary material for: Postpartum hemorrhage care bundles to improve adherence to guidelines: A WHO technical consultation
Source: Int J Gynaecol Obstet. 2019 Dec 23;148(3):290–9. doi: 10.1002/ijgo.13028 (PMC7064978; doi:10.1002/ijgo.13028)
Supplement: Supplementary file 6 — File S2. Methodologic details. [file IJGO-148-290-s006.docx]

# **Supplementary File S2** Methodologic details.

## *Systematic search of the literature strategy*

A bibliographic search was carried out on PubMed looking for designs and implementations of bundles, and with Internet general search engines, looking for non-indexed grey literature. Bibliographic search about perinatal bundles was carried out on PubMed, using thesaurus and terms of the natural language, with maximum sensibility, and with general search engines looking for grey literature. The following combinations of medical subject headings (MeSH) and free text terms were utilized:

(Patient Care Bundles[MeSH] OR Care Bundle*[ti] OR Safety Bundle*[ti]) OR (Evidence Based[tiab] AND Bundle[tiab])

We also conducted a literature search for Maternal and Perinatal Bundles: (Patient Care Bundles[MeSH] OR Care Bundle*[tiab] OR Safety Bundle*[tiab] OR Consensus Bundle*[tiab]) AND (Perinatal Care[Mesh] OR Perinatal[tiab] OR Obstetric*[tiab] OR Maternal[tiab] OR Pregnancy[Mesh] OR Pregnan*[tiab] OR Labor[tiab] OR Labour[tiab] OR Prenatal Care[Mesh] OR Prenatal[tiab] OR Antenatal[tiab] OR Ante Natal[tiab] OR Delivery, Obstetric[Mesh] OR Postpartum Period[Mesh] OR Puerper*[tiab] OR Postpartum[tiab] OR Post Partum[tiab] OR Postnatal Care[Mesh] OR Postnatal[tiab] OR Post Natal[tiab] OR Parturition[Mesh] OR Parturition[tiab] OR Birth[tiab] OR Chilbirth[tiab])

And PPH Bundles: (Patient Care Bundles[MeSH] OR Care Bundle*[tiab] OR Safety Bundle*[tiab] OR Consensus Bundle*[tiab]) AND (Postpartum Hemorrhage[Mesh] OR Postpartum Hemorrhage*[tiab] OR PPH[tiab] OR Post-Partum Hemorrage*[tiab] OR Postpartum Haemorrhage[tiab] OR Post-Partum Haemorrhage[tiab] OR Obstetrical Hemorrhage*[tiab] OR Obstetrical Haemorrhage*[tiab] OR Obstetric Hemorrhage*[tiab] OR Obstetric Haemorrhage*[tiab] OR ((Labor, Obstetric[Mesh] OR Labor[tiab] OR Labour[tiab] OR Partum[tiab] OR Postpartum[tiab]) AND (Hemorrhage[Mesh] OR Hemorrhage*[tiab] OR Haemorrhage*[tiab])))

For general care bundles, the literature search identified 648 potentially relevant papers. Title and abstracts were screened to identify relevant studies. National and international guidelines, randomized controlled trials (RCTs), systematic reviews, reviews, quasi-experimental studies, quality improvement studies and references of the included articles that provided a definition or described the methodology used for the development of bundles regardless of the area under study were eligible for inclusion.

**Selection of relevant papers**

Initially studies were reviewed by title. After excluding non-relevant studies (payment bundles, non English or Spanish publications, duplicates, other topic and/or no full text), 594 full-text papers were further screened. After exclusion of texts that were editorials, study protocols, comments, single case study, or letters, 560 articles were assessed for eligibility.

Of these papers, 315 were included according to the exclusion criteria shown in Supplementary Figure 1.

***Consensus Method***

We used a three-stage modified Delphi method, starting with two rounds of individual and anonymous online questionnaires that consisted of closed-ended and open-ended questions, followed by a third round: an in-person group discussion.

The modified Delphi method was selected because this technique synthesizes the knowledge of a large number of individuals – across diverse settings, locations, and areas of expertise – participating anonymously to avoid the consensus process being dominated by a few experts. The experts provided individual input through successive questionnaires that were designed by the study’s organizers. Experts’ responses were analyzed in statistical format, which reflects the degree of agreement reached. Online rounds were based on web-based questionnaires (Survey Monkey™). For each questionnaire, a second round was conducted as consensus was not achieved in the first round. During subsequent rounds, experts received the overall rating distributions and comments of the preceding rounds in order to build consensus.

***Rating and Weighing***

Multi-criteria Decision Analysis (MCDA) was used for rating and weighing the criteria used to guide the selection of bundle interventions. MCDA is a widely used technique that supports decision making when numerous conflicting evaluations are being assessed (Devlin 2011, Thokala 2012). Measurements in MCDA are derived subjectively, as indicators of the strength of various preferences.

For this study, experts were asked to rate criteria and bundles on a 9-point Likert RAND Appropriateness Scale (RAS) (Flitch 2001). Then, overall median rating were calculated. Median rating of 1-3 points were classified low; median rating of 4-6 points were classified intermediate; median rating of 7-9 points were classified high.

Next, a mathematical equation was used to estimate a weighted rating for each care bundle. The criteria were weighted to demonstrate their relative importance to each other, and bundles were rated to reflect their performance against each criterion. These ratings and weights were used to generate an overall “weighted benefit rating” for each care bundle. These overall values established a preference order of alternative bundles (Thokala 2011). The ﬁrst step in this approach was to rate the performance levels of each bundle on each criterion (value rating). Then, a partial weighted rating was calculated by multiplying the median value ratings for each criterion by its weight. Finally, additive aggregation (weighted sum), the most common value-measurement modelling approach, was applied, based on the following equation:


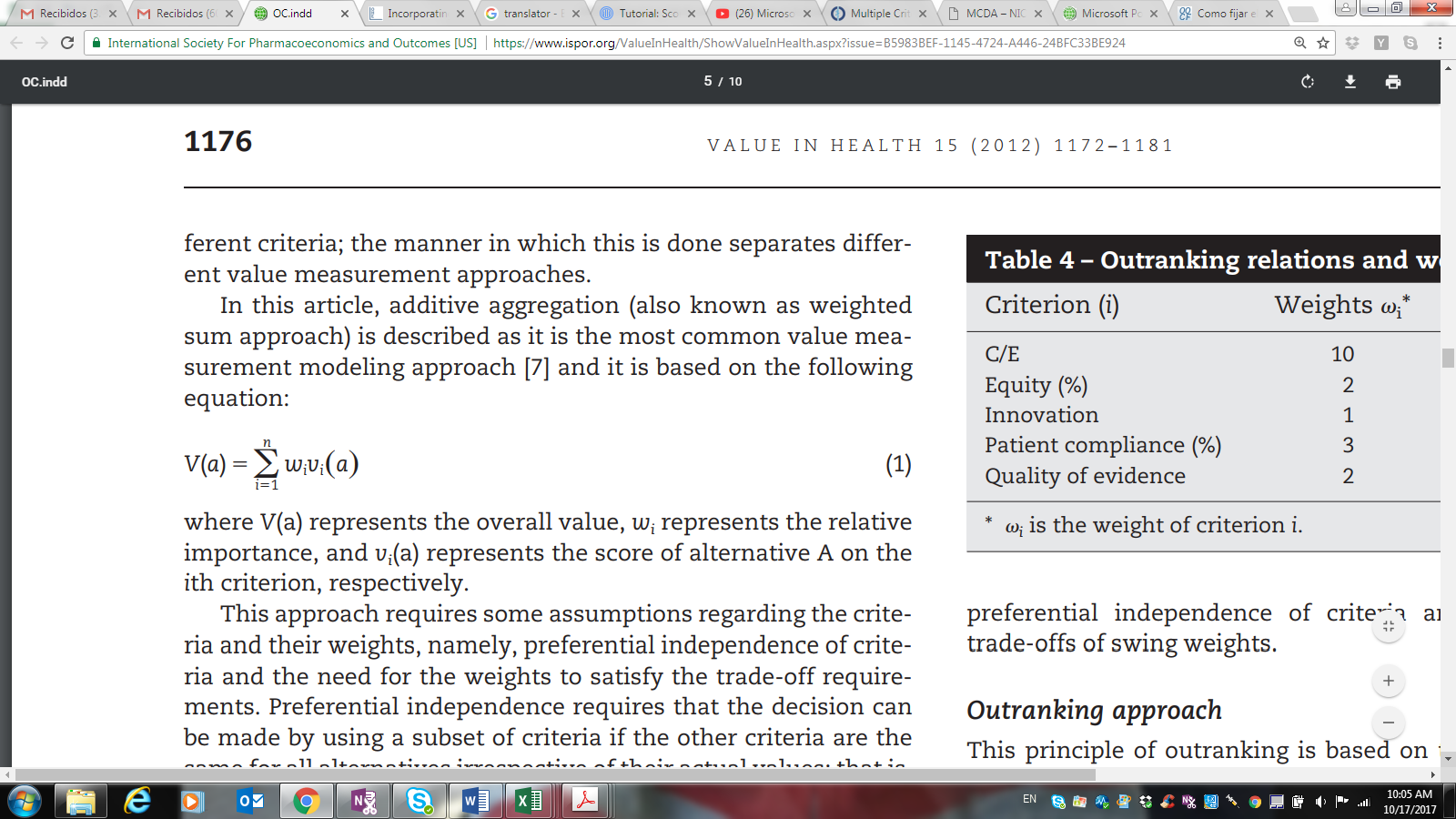


where V (a) represents the overall value, ωi represents the relative importance (weight), and vi (a) represents the rating of bundle A for it criterion.

***Analysis of Disagreements***

For the measurement of disagreement, the RAND DI (Disagreement Index) was applied. DI is composed of two major components: (i) the inter-percentile range (IPR) and (ii) the inter-percentile range adjusted for symmetry (IPRAS). The IPRAS was calculated with the following equation: IPRAS = IPRr + (AI * CFA) , where IPRr is the inter-percentile range required for disagreement when there is perfect symmetry (constant of 2.4); the AI is the asymmetry index, which is the distance between the center point of the IPR and the center point of a 9-point scale; and CFA is the correction factor for asymmetry (constant set at 1.5). The RAND DI was calculated with the following equation:

DI = IPR/IPRAS.

If the DI for an item was > 1.0, it indicated disagreement on the ratings. If the DI was < 1.0 (including negative values
