# Supplementary material for: Postpartum hemorrhage care bundles to improve adherence to guidelines: A WHO technical consultation
Source: Int J Gynaecol Obstet. 2019 Dec 23;148(3):290–9. doi: 10.1002/ijgo.13028 (PMC7064978; doi:10.1002/ijgo.13028)
Supplement: Supplementary file 7 — File S3. Survey 1. [file IJGO-148-290-s007.docx]

**Supplementary File S3 Survey 1**

**Domain 1: Effects of interventions**

**1.1 Desirable effects**

How substantial are the desirable anticipated effects of the intervention?

The more substantial the desirable effects, the more likely it is that an intervention will be selected. Judgments about how substantial effects are should take into account the absolute magnitude of the effect (such as the proportion of people who would benefit) and the importance of the outcome (such as how much it is valued by the people affected).

If evidence suggests that the intervention will have a substantial desirable effect, then the intervention will score high. On the contrary, if the effect was very little or there was no desirable effect, then the intervention will score low.

a) Do you consider this criterion relevant in order to select interventions to be included in care bundles?

Yes No

b) Regardless of whether you have included or excluded this criterion, if this criterion were to be included, how would you rate its relevance? (1=not relevant; 9=critical)

1 2 3 4 5 6 7 8 9

c) Would you like to add any comment about this criterion?

**1.2 Undesirable effects**

How substantial are the undesirable anticipated effects of the intervention?

The more substantial the undesirable effects (including the relative burden of interventions), the less likely it is that an intervention should be selected. Judgments about how substantial undesirable effects are should take into account the absolute magnitude of the effect (such as the proportion of people who would benefit) and the importance of the outcome (such as how much it is valued by the people affected). If evidence suggests that the intervention will have a substantial undesirable effect, then the intervention will score low. On the contrary, if the undesirable effect is small or if there was no undesirable effect, then the intervention will score high.

a) Do you consider this criterion relevant in order to select interventions to be included in care bundles?

Yes No

b) Regardless of whether you have included or excluded this criterion, if this criterion were to be included, how would you rate its relevance? (1=not relevant; 9=critical)

1 2 3 4 5 6 7 8 9

c) Would you like to add any comment about this criterion?

**1.3 Certainty of the Evidence**

What is the overall certainty (also called quality) of the evidence of the intervention’s effects?

In the context of making decisions, the certainty rating reflects the extent of our confidence that the estimate of an effect (including test accuracy and associations) is adequate to support a particular selection. The higher the certainty of the evidence that indicates that there are benefits across outcomes, the higher the likelihood that the intervention will be selected. The less certain the evidence is for the main outcomes (desirable and undesirable effects, including the burden), the less likely it is that a strong recommendation should be made for an intervention to be selected.

a) Do you consider this criterion relevant in order to select interventions to be included in care bundles?

Yes No

b) Regardless of whether you have included or excluded this criterion, if this criterion were to be included, how would you rate its relevance? (1=not relevant; 9=critical)

1 2 3 4 5 6 7 8 9

c) Would you like to add any comment about this criterion?

**1.4 Values and preferences**

Is there significant uncertainty about, or variability in, how much women value the outcomes associated with the intervention?

Uncertainty about how much those affected (patients or their carers) value the outcomes

of interest can be a reason for not selecting an intervention. Variability in how patients value the main outcomes (to the extent that individuals with different values would make different decisions) is another reason for not selecting an intervention.

a) Do you consider this criterion relevant in order to select interventions to be included in care bundles?

Yes No

b) Regardless of whether you have included or excluded this criterion, if this criterion were to be included, how would you rate its relevance? (1=not relevant; 9=critical)

1 2 3 4 5 6 7 8 9

c) Would you like to add any comment about this criterion?

**1.5 Balance of effects**

Does the balance between desirable and undesirable effects favour the intervention?

Judgments about the balance between the desirable and undesirable effects need to take into account the preceding four criteria: the magnitude of the desirable and undesirable effects, the certainty of the evidence supporting the anticipated effects, and how much those affected value the outcomes.

a) Do you consider this criterion relevant in order to select interventions to be included in care bundles?

Yes No

b) Regardless of whether you have included or excluded this criterion, if this criterion were to be included, how would you rate its relevance? (1=not relevant; 9=critical)

1 2 3 4 5 6 7 8 9

c) Would you like to add any comment about this criterion?

**Domain 2: Resources**

**2.1. Resource required**

How large are the resource requirements (costs – both money and time required) of the intervention?

The greater the cost, the less likely it is that an intervention will be selected.

a) Do you consider this criterion relevant in order to select interventions to be included in care bundles?

Yes No

b) Regardless of whether you have included or excluded this criterion, if this criterion were to be included, how would you rate its relevance? (1=not relevant; 9=critical)

1 2 3 4 5 6 7 8 9

c) Would you like to add any comment about this criterion?

**2.2 Certainty of evidence on required resources**

What is the certainty of the evidence for the costs of the intervention?

If resource use is considered critical for a recommendation, the less certain the evidence for resource requirements, the less likely it is that a panel should select or not the intervention. Judgments about the certainty of the evidence for resource requirements are similar to judgments about the evidence of effects.

a) Do you consider this criterion relevant in order to select interventions to be included in care bundles?

Yes No

b) Regardless of whether you have included or excluded this criterion, if this criterion were to be included, how would you rate its relevance? (1=not relevant; 9=critical)

1 2 3 4 5 6 7 8 9

c) Would you like to add any comment about this criterion?

**2.3 Cost effectiveness**

Is the intervention cost effective?

The greater the cost in relation to the net benefit, the less likely it is that an intervention should be selected. Judgments about the cost effectiveness of an intervention need to take into account several criteria, including:

- The balance between the desirable and undesirable effects (the net benefit), the certainty of the evidence of effects, and uncertainty about or variability in how much people value the main outcomes
- Resource requirements (cost) and uncertainty about the costs.

a) Do you consider this criterion relevant in order to select interventions to be included in care bundles?

Yes No

b) Regardless of whether you have included or excluded this criterion, if this criterion were to be included, how would you rate its relevance? (1=not relevant; 9=critical)

1 2 3 4 5 6 7 8 9

c) Would you like to add any comment about this criterion?

**Domain 3: Equity**

What would be the impact of the intervention on health equity?

This criterion evaluates if an intervention is expected to reduce health inequities. It considers whether an intervention will reduce differences in the effectiveness for disadvantaged populations within countries, such as low income groups, less educated, and/or rural populations, etc. For example an effective intervention implementable at all levels of care that may not require out-of-pocket expenditures from women, may reduce inequalities. Conversely, selecting an intervention that can only be implemented at referral hospitals may increase inequalities in settings where access to complex care is limited for disadvantaged or rural populations.

a) Do you consider this criterion relevant in order to select interventions to be included in care bundles?

Yes No

b) Regardless of whether you have included or excluded this criterion, if this criterion were to be included, how would you rate its relevance? (1=not relevant; 9=critical)

1 2 3 4 5 6 7 8 9

c) Would you like to add any comment about this criterion?

**Domain 4: Acceptability**

Is the intervention acceptable to key stakeholders (women and providers)?

The less acceptable an intervention is to key stakeholders, the less likely it is to be included, or if it is included, the more likely it is that an implementation strategy might be needed to address concerns about acceptability.

An intervention might vary on its acceptability level due to ethical principles (such as autonomy, beneficence or justice), as well as the distribution of the desirable and undesirable effects and costs (who benefits or is harmed and who pays or saves).

a) Do you consider this criterion relevant in order to select interventions to be included in care bundles?

Yes No

b) Regardless of whether you have included or excluded this criterion, if this criterion were to be included, how would you rate its relevance? (1=not relevant; 9=critical)

1 2 3 4 5 6 7 8 9

c) Would you like to add any comment about this criterion?

**Domain 5: Feasibility**

Is the intervention feasible to implement?

Feasibility is influenced by factors such as the resources available, infrastructure and training. If the intervention is not already in use, this criterion evaluates if it can be introduced with a reasonable investment of cost, time and training. The less feasible (capable of being accomplished or brought about) an intervention is, the less likely it is that it should be selected. Clinicians might find a care bundle unhelpful if the included interventions are not implementable in their settings

In other words, this criterion evaluates if the intervention can be achieved during routine clinical practice with the resources, infrastructure and training already available, or with a minimum increase of extra resources.

a) Do you consider this criterion relevant in order to select interventions to be included in care bundles?

Yes No

b) Regardless of whether you have included or excluded this criterion, if this criterion were to be included, how would you rate its relevance? (1=not relevant; 9=critical)

1 2 3 4 5 6 7 8 9

c) Would you like to add any comment about this criterion?

**Domain 6: Indicator Measurability**

This criterion evaluates whether a quantitative indicator for the intervention’s use is available and can be simply and reliably measured during routine clinical practice, without extra resources or with a minimum of extra resources.

According to this criterion, interventions with indicators that can be easily and reliably collected at the patient level during routine clinical care will be scored high; while those interventions with indicators that require exceptional resources to measure/collect will get a low score.

Indicators are quantitative or qualitative factors or variables that provide a simple and reliable means to measure achievement, to reflect the change connected to an intervention, or to help assess performance.

a) Do you consider this criterion relevant in order to select interventions to be included in care bundles?

Yes No

b) Regardless of whether you have included or excluded this criterion, if this criterion were to be included, how would you rate its relevance? (1=not relevant; 9=critical)

1 2 3 4 5 6 7 8 9

c) Would you like to add any comment about this criterion?

Thank you for participating in this survey
