# Supplementary material for: Postpartum hemorrhage care bundles to improve adherence to guidelines: A WHO technical consultation
Source: Int J Gynaecol Obstet. 2019 Dec 23;148(3):290–9. doi: 10.1002/ijgo.13028 (PMC7064978; doi:10.1002/ijgo.13028)
Supplement: Supplementary file 8 — File S4. Survey 2. [file IJGO-148-290-s008.docx]

**Supplementary File S4 Survey 2**

**Bundle for PPH Prevention and Recognition**

This bundle should be initiated for every woman giving birth, at either the 3^rd^ stage or during the first 24 hours postpartum.

| **Intervention** | **Description** |
| --- | --- |
| **1. Uterotonic drugs** | - 1. The preferred drug for prevention of PPH is Oxytocin (10 IU, IV/IM) (WHO, 2012)   2. If unavailable, give IM ergometrine/methylergometrine or the fixed drug combination of oxytocin and ergometrine, if not contraindicated (WHO, 2012)   3. If IM or IV uterotonics are unavailable, give oral misoprostol (600 micrograms) orally (WHO, 2012) |
| **2. Controlled Cord Traction** | After the birth of the baby and it is assessed that there are no other babies in utero, gentle traction is applied to the umbilical cord with one hand, while the other hand applies abdominal counter-pressure on the uterus. |
| **3. Postpartum abdominal uterine tonus assessment** | Cupping one hand on the uterine fundus with the other hand on the bottom of the uterus to brace it, massage in order to assess uterine firmness, if the uterus is soft or flabby this may indicate uterine atony |

1. How would you rate this bundle to be implemented in **community setting (home births)** according to each criteria?

| Criteria | Scale description | Rating |
| --- | --- | --- |
| Equity | 1=increase inequalities substantially;  9=reduce inequalities substantially | 1 2 3 4 5 6 7 8 9 |
| Acceptability | 1=Hardly accepted by stakeholders;  9=Highly accepted by stakeholders | 1 2 3 4 5 6 7 8 9 |
| Feasibility | 1=Hardly implementable;  9=Highly implementable | 1 2 3 4 5 6 7 8 9 |
| Indicator Measurability | 1=Indicators hardly available;  9=Indicators easily available | 1 2 3 4 5 6 7 8 9 |
| No or Minimal Extra Resources Required | 1=Substantial extra resources;  9=No extra resources | 1 2 3 4 5 6 7 8 9 |

2. How would you rate this bundle to be implemented in **primary health care centers** according to each criteria?

| Criteria | Scale description | Rating |
| --- | --- | --- |
| Equity | 1=increase inequalities substantially;  9=reduce inequalities substantially | 1 2 3 4 5 6 7 8 9 |
| Acceptability | 1=Hardly accepted by stakeholders;  9=Highly accepted by stakeholders | 1 2 3 4 5 6 7 8 9 |
| Feasibility | 1=Hardly implementable;  9=Highly implementable | 1 2 3 4 5 6 7 8 9 |
| Indicator Measurability | 1=Indicators hardly available;  9=Indicators easily available | 1 2 3 4 5 6 7 8 9 |
| No or Minimal Extra Resources Required | 1=Substantial extra resources;  9=No extra resources | 1 2 3 4 5 6 7 8 9 |

3. How would you rate this bundle to be implemented in **hospitals (all levels)** according to each criteria?

| Criteria | Scale description | Rating |
| --- | --- | --- |
| Equity | 1=increase inequalities substantially;  9=reduce inequalities substantially | 1 2 3 4 5 6 7 8 9 |
| Acceptability | 1=Hardly accepted by stakeholders;  9=Highly accepted by stakeholders | 1 2 3 4 5 6 7 8 9 |
| Feasibility | 1=Hardly implementable;  9=Highly implementable | 1 2 3 4 5 6 7 8 9 |
| Indicator Measurability | 1=Indicators hardly available;  9=Indicators easily available | 1 2 3 4 5 6 7 8 9 |
| No or Minimal Extra Resources Required | 1=Substantial extra resources;  9=No extra resources | 1 2 3 4 5 6 7 8 9 |

**Bundle for First Response Management of PPH.**

This bundle should be initiated with excessive bleeding, as soon as it is identified, at either the 3^rd^ stage or during the first 24 hours postpartum.

| **Intervention** | **Description** |
| --- | --- |
| **1. Uterotonic drugs** | Oxytocin IV is the first choice. Starting dose: 20IU (diluted in 500 ml of isotonic crystalloids IV, in 30 min). Continuing dose: 20IU (diluted in 500 ml of isotonic crystalloids IV, in 60 min).   1. If oxytocin unavailable, IV ergot alkaloids 0.2 mg or injectable prostaglandins, i.e. Carboprost can be administered intramyometrially or intramuscularly in a dose of 0.25 mg; this dose can be repeated every 15 minutes for a total dose of 2 mg. 2. Misoprostol 800 micrograms sublingually. |
| **2. Isotonic crystalloids (IV)*** | Starting dose: 500 ml of isotonic crystalloids IV, in 30 min  Continuing dose: 500 ml of isotonic crystalloids IV, in 60 min |
| **3. Tranexamic Acid (TXA)** | A fixed dose of TXA 1 g (100mg/ml) intravenously (IV) at 1 ml per minute, within 3 hours of the time of diagnosis (if unknown, time of birth) |
| **4. Uterine Massage** | If the uterine tone assessment shows that the uterus is soft or flabby, cup one hand on the uterine fundus with the other hand on the bottom of the uterus to brace it, then massage the fundus in a circular manner, continue to massage until the uterus is firm |
| Comments: If the PPH is in the context of a placental retention, the placenta should be extracted (see specific WHO guideline for placental retention), and a single dose of antibiotics administered. | |

***** The initial fluid resuscitation is performed together with IV uterotonics. If IV uterotonics are not available, fluid resuscitation should be started in parallel to sublingual misoprostol or other parenteral uterotonic.

1. How would you rate this bundle to be implemented in **community setting (home births)** according to each criteria?

| Criteria | Scale description | Rating |
| --- | --- | --- |
| Equity | 1=increase inequalities substantially;  9=reduce inequalities substantially | 1 2 3 4 5 6 7 8 9 |
| Acceptability | 1=Hardly accepted by stakeholders;  9=Highly accepted by stakeholders | 1 2 3 4 5 6 7 8 9 |
| Feasibility | 1=Hardly implementable;  9=Highly implementable | 1 2 3 4 5 6 7 8 9 |
| Indicator Measurability | 1=Indicators hardly available;  9=Indicators easily available | 1 2 3 4 5 6 7 8 9 |
| No or Minimal Extra Resources Required | 1=Substantial extra resources;  9=No extra resources | 1 2 3 4 5 6 7 8 9 |

2. How would you rate this bundle to be implemented in **primary health care centers** according to each criteria?

| Criteria | Scale description | Rating |
| --- | --- | --- |
| Equity | 1=increase inequalities substantially;  9=reduce inequalities substantially | 1 2 3 4 5 6 7 8 9 |
| Acceptability | 1=Hardly accepted by stakeholders;  9=Highly accepted by stakeholders | 1 2 3 4 5 6 7 8 9 |
| Feasibility | 1=Hardly implementable;  9=Highly implementable | 1 2 3 4 5 6 7 8 9 |
| Indicator Measurability | 1=Indicators hardly available;  9=Indicators easily available | 1 2 3 4 5 6 7 8 9 |
| No or Minimal Extra Resources Required | 1=Substantial extra resources;  9=No extra resources | 1 2 3 4 5 6 7 8 9 |

3. How would you rate this bundle to be implemented in **hospitals (all levels)** according to each criteria?

| Criteria | Scale description | Rating |
| --- | --- | --- |
| Equity | 1=increase inequalities substantially;  9=reduce inequalities substantially | 1 2 3 4 5 6 7 8 9 |
| Acceptability | 1=Hardly accepted by stakeholders;  9=Highly accepted by stakeholders | 1 2 3 4 5 6 7 8 9 |
| Feasibility | 1=Hardly implementable;  9=Highly implementable | 1 2 3 4 5 6 7 8 9 |
| Indicator Measurability | 1=Indicators hardly available;  9=Indicators easily available | 1 2 3 4 5 6 7 8 9 |
| No or Minimal Extra Resources Required | 1=Substantial extra resources;  9=No extra resources | 1 2 3 4 5 6 7 8 9 |

**Bundle for Clinical management of refractory PPH**

This bundle should be initiated if bleeding continues after 30 min from the first response, at either the 3^rd^ stage or during the first 24 hours postpartum.

| **Intervention** | **Description** |
| --- | --- |
| **1. Uterotonic drugs** | Oxytocin IV is the first choice. Starting dose: 20IU (diluted in 500 ml of isotonic crystalloids IV, in 30 min). Continuing dose: 20IU (diluted in 500 ml of isotonic crystalloids IV, in 60 min).   1. If oxytocin unavailable, IV ergot alkaloids 0.2 mg or injectable prostaglandins, i.e. Carboprost can be administered intramyometrially or intramuscularly in a dose of 0.25 mg; this dose can be repeated every 15 minutes for a total dose of 2 mg. 2. Misoprostol 800 micrograms sublingually. |
| **2. Isotonic crystalloids (IV)*** | Starting dose: 500 ml of isotonic crystalloids IV, in 30 min  Continuing dose: 500 ml of isotonic crystalloids IV, in 60 min |
| **3. Tranexamic Acid (TXA)** | A second dose of TXA 1 g (100mg/ml) intravenously (IV) at 1 ml per minute, given 30 minutes from the first dose. |
| **4. Intrauterine Balloon Tamponade (IBT)**** | The procedure entails insertion of a deflated/uninflated balloon into the uterine cavity and then inflating it to achieve a tamponade effect. |
| **5. Non-pneumatic anti shock Garment** | Used as a temporizing measure until source of bleeding found and treated. NASG is a lower body compression device made of stretch neoprene which closes tightly with Velcro in segments for the ankles, calves, thighs, pelvis, and abdomen and is applied rapidly starting at the ankles. |

***** The initial fluid resuscitation is performed together with IV uterotonics. If IV uterotonics are not available, fluid resuscitation should be started simultaneously with sublingual misoprostol or other parenteral uterotonic.

** In setting where this is not feasible, bimanual uterine compression or external aortic compression are alternative temporizing measures.

1. How would you rate this bundle to be implemented in **community setting (home births)** according to each criteria?

| Criteria | Scale description | Rating |
| --- | --- | --- |
| Equity | 1=increase inequalities substantially;  9=reduce inequalities substantially | 1 2 3 4 5 6 7 8 9 |
| Acceptability | 1=Hardly accepted by stakeholders;  9=Highly accepted by stakeholders | 1 2 3 4 5 6 7 8 9 |
| Feasibility | 1=Hardly implementable;  9=Highly implementable | 1 2 3 4 5 6 7 8 9 |
| Indicator Measurability | 1=Indicators hardly available;  9=Indicators easily available | 1 2 3 4 5 6 7 8 9 |
| No or Minimal Extra Resources Required | 1=Substantial extra resources;  9=No extra resources | 1 2 3 4 5 6 7 8 9 |

2. How would you rate this bundle to be implemented in **primary health care centers** according to each criteria?

| Criteria | Scale description | Rating |
| --- | --- | --- |
| Equity | 1=increase inequalities substantially;  9=reduce inequalities substantially | 1 2 3 4 5 6 7 8 9 |
| Acceptability | 1=Hardly accepted by stakeholders;  9=Highly accepted by stakeholders | 1 2 3 4 5 6 7 8 9 |
| Feasibility | 1=Hardly implementable;  9=Highly implementable | 1 2 3 4 5 6 7 8 9 |
| Indicator Measurability | 1=Indicators hardly available;  9=Indicators easily available | 1 2 3 4 5 6 7 8 9 |
| No or Minimal Extra Resources Required | 1=Substantial extra resources;  9=No extra resources | 1 2 3 4 5 6 7 8 9 |

3. How would you rate this bundle to be implemented in **hospitals (all levels)** according to each criteria?

| Criteria | Scale description | Rating |
| --- | --- | --- |
| Equity | 1=increase inequalities substantially;  9=reduce inequalities substantially | 1 2 3 4 5 6 7 8 9 |
| Acceptability | 1=Hardly accepted by stakeholders;  9=Highly accepted by stakeholders | 1 2 3 4 5 6 7 8 9 |
| Feasibility | 1=Hardly implementable;  9=Highly implementable | 1 2 3 4 5 6 7 8 9 |
| Indicator Measurability | 1=Indicators hardly available;  9=Indicators easily available | 1 2 3 4 5 6 7 8 9 |
| No or Minimal Extra Resources Required | 1=Substantial extra resources;  9=No extra resources | 1 2 3 4 5 6 7 8 9 |
