# Supplementary material for: Postpartum hemorrhage care bundles to improve adherence to guidelines: A WHO technical consultation
Source: Int J Gynaecol Obstet. 2019 Dec 23;148(3):290–9. doi: 10.1002/ijgo.13028 (PMC7064978; doi:10.1002/ijgo.13028)
Supplement: Supplementary file 9 — File S5. Technical consultation agenda. [file IJGO-148-290-s009.docx]

**Supplementary File S5 Technical consultation agenda**

**World Health Organization**

**Technical Consultation on Care Bundles for PPH**

**December 7 – 8, 2017**

**Agenda**

**Meeting objectives**

1. Define care bundles for PPH that are feasible [including low resource settings]
2. Define the most important steps needed to achieve effective implementation

3. Determine the research and implementation research that is required to create the evidence base to implement care bundles for PPH and transition them to scale.

| **Day 1 – Dec 7, 2017** | | |
| --- | --- | --- |
| 8.00h-8.30 | Registration |  |
| 8.30h-8.50h | Opening   - Welcome - Introductions - Declaration of Interests - Appointment of the chairperson and the meeting facilitator | JPS, JL, PL, JH |
| 8.50h-10:00h | Background and Motivation for Developing Care Bundles for PPH | JPS |
| 10.00h-10.30h | Coffee break |  |
| 10.30h-12.15h | Process of assembling PPH Bundles  Pre-meeting consultations | FA |
| 12.15h-13.15h | Lunch break |  |
| 13.15h-13.45h | PPH Prevention Bundles  Should we have a PPH prevention bundle? | JPS |
| 13.45h-15.15h | PPH First Response Bundles   - Hospitals - PHC - Community health services | SM/JPS |
| 15.15h-15.45h | Coffee break |  |
| 15.45h-17.00h | PPH Bundles for refractory bleeding   - Hospitals - PHC - Community health services | JPS |
| 17.00 h | Closure of Day 1 | PL |
| 17.30 | Reception and Dinner at Cinquecento –  500 Harrison Ave, Boston, MA 02118 |  |

| **Day 2 – Dec 8, 2017** | | |
| --- | --- | --- |
| 8.30h-9.00h | Summary of the previous day and recap on the proposed bundles | TB |
| 9.00-10.30h | Is it worth pursuing the “Bundles approach” for reducing PPH related deaths and morbidities?  Discussion on implementation aspects of proposed PPH bundles (Hospitals and PHC)   - Anticipated problems - Mitigation strategy - Perception of anticipated benefits vs harms | SM |
| 10.30h-11.00h | Coffee break |  |
| 11.00h-12.30h | Is it worth pursuing the “Bundles approach” for reducing PPH related deaths and morbidities? (Part 2)  Discussion on implementation aspects of proposed PPH bundles (Community settings)   - Anticipated problems - Mitigation strategy - Perception of anticipated benefits vs harms | JPS |
| 12.30h-13.30h | Lunch break |  |
| 13.30h-14.30h | What is the research and implementation research needed to implement and transition to scale Care Bundles for PPH?   - Is effectiveness research needed? - Is an assessment of barriers and facilitators needed? - Focus on implementation research only? | FA |
| 14.30h-15.30h | What are the necessary steps to achieve effective implementation? | JL |
| 15.30-16:00h | Coffee break |  |
| 16.00-16.30h | Meeting report and publications | JPS/SM/FA |
| 16.30-17.00h | Next steps and meeting closure | PL |
