# Supplementary material for: Postpartum hemorrhage care bundles to improve adherence to guidelines: A WHO technical consultation
Source: Int J Gynaecol Obstet. 2019 Dec 23;148(3):290–9. doi: 10.1002/ijgo.13028 (PMC7064978; doi:10.1002/ijgo.13028)
Supplement: Supplementary file 10 — Box S1. Examples of best clinical practices. [file IJGO-148-290-s010.docx]

**Supplementary Box S1** Examples of best clinical practices.

**PPH Prevention**

* Anemia prevention, detection, and control

*Malaria prevention, detection, and control

*HIV status and treatment if necessary

* Identification of previous and current co-morbidities that may impact hemorrhage, manage co-morbidities, and refer to specialist as necessary

*Identification of bleeding in the current pregnancy or bleeding/PPH in a previous pregnancy

*Birth planning and complication preparedness

*Prophylactic uterotonics between birth of baby and delivery of placenta

**First Response to PPH: in addition to bundles**

*Early recognition of excessive bleeding

* Calling for assistance

*Reassurance to woman and her family that everything is being done

*Monitor vital signs (pulse, BP, temperature, and respirations), volume and rate of blood flow, level of consciousness or anxiety, skin color, and response to treatments

*Catheterize the bladder or have the woman void

*Determine cause of PPH

* Check status of placenta:

Is placenta retained?

If placenta delivered, was it intact?

*Examine cervix, vagina, and perineum for tears

* Palpate uterus for firmness and massage if not firm

* Rule out etiologies besides atony or retained placenta

*Laboratory Testing for Hgb/HCT, clotting factors (or bedside clotting test), and Type and Cross match

*Referral and Transfer to higher level facility capable of blood transfusions/surgery

**Management of Continuing PPH: in addition to bundles**

*If not already done, Laboratory Testing for Hgb/HCT, clotting factors (or bedside clotting test), and Type and Cross match

*Order blood

*Inform woman and her family of treatment options, provide reassurance

*Continue monitoring for status and development of shock, if shock suspected begin shock management

*Begin blood/blood products transfusion based on laboratory results (or bedside clotting test)

*Surgical Management

*Begin with conservative management, try to spare the uterus, e.g.

**Uterine compression sutures

**Uterine or utero-ovarian artery ligation

*If woman continues to bleed, perform subTotal (supracervical) or total hysterectomy

Adapted from: WHO 2017: Managing complications in pregnancy and childbirth: a guide for midwives and doctors – 2nd ed. ISBN 978-92-4-156549-3 .
